# Exosomal miRNAs and miRNA dysregulation in cancer-associated fibroblasts

**DOI:** 10.1186/s12943-017-0718-4

**Published:** 2017-08-29

**Authors:** Fengming Yang, Zhiqiang Ning, Ling Ma, Weitao Liu, Chuchu Shao, Yongqian Shu, Hua Shen

**Affiliations:** 1 Department of Oncology, Sir Run Run Hospital, Nanjing Medical University, Nanjing, China; 2 Department of Oncology, The first People’s Hospital of Wujiang district, Suzhou, 215200 China; 3 Department of Oncology, The First Affiliated Hospital of Nanjing Medical University, 300 Guangzhou Road, Nanjing, 210029 People’s Republic of China; 4 Jiangsu Key Lab of Cancer Biomarkers, Prevention and Treatment, Collaborative Innovation Center for Cancer Personalized Medicine, Shanghai, China; 5 Department of Pathology, Nanjing Medical University, Nanjing, People’s Republic of China

**Keywords:** Cancer-associated fibroblasts, MiRNA, Exosome, Cancer, Mechanism

## Abstract

**Purpose:**

The present review aimed to assess the role of exosomal miRNAs in cancer-associated fibroblasts (CAFs), normal fibroblasts (NFs), and cancer cells. The roles of exosomal miRNAs and miRNA dysregulation in CAF formation and activation were summarized.

**Methods:**

All relevant publications were retrieved from the PubMed database, with key words such as CAFs, CAF, stromal fibroblasts, cancer-associated fibroblasts, miRNA, exosomal, exosome, and similar terms.

**Results:**

Recent studies have revealed that CAFs, NFs, and cancer cells can secrete exosomal miRNAs to affect each other. Dysregulation of miRNAs and exosomal miRNAs influence the formation and activation of CAFs. Furthermore, miRNA dysregulation in CAFs is considered to be associated with a secretory phenotype change, tumor invasion, tumor migration and metastasis, drug resistance, and poor prognosis.

**Conclusions:**

Finding of exosomal miRNA secretion provides novel insights into communication among CAFs, NFs, and cancer cells. MicroRNA dysregulation is also involved in the whole processes of CAF formation and function. Dysregulation of miRNAs in CAFs can affect the secretory phenotype of the latter cells.

## Background

MicroRNAs (miRNAs), a major class of small non-coding RNAs that mediate post-transcriptional gene silencing by binding to the 3′-untranslated region (UTR) or open reading frames (ORFs) of target mRNAs, have been widely studied in various physiological and pathological processes [1, 2]. Similar to protein coding genes, most miRNA genes are transcribed by RNA polymerase II to form terminal and poly-adenylated RNA precursors or primary transcripts (pri-miRNAs) [3]. Stress-signals in cancer cells regulate the production of particular pri-miRNAs by controlling the functionality of certain transcription factors [4]. Established roles of miRNAs include the regulation of cell growth and tissue differentiation [5]. As both processes are dysregulated in cancer formation and development, miRNAs can participate in several processes of cancer, such as metastasis, tumorigenesis, and drug resistance [6–8]. Previous studies reported that miRNAs might serve as a tool for cancer diagnosis [9], and dysregulation of miRNA expression could be used for patient prognosis [10]. In recent years, with the progress witnessed in exosome assessment, use of miRNA as a biomarker or therapeutic tool in clinical practice has become a reality [11, 12].

The microenvironment consists of different types of normal cells, including fibroblasts, endothelial cells, pericytes, immune cells, and local or bone marrow-derived stromal stem and progenitor cells, and the surrounding extracellular matrix (ECM) [13]. Tumor microenvironment heterogeneity can influence tumorigenesis, invasion, metastasis, and the therapeutic response [14, 15]. Cancer-associated fibroblasts (CAFs) are vital constituents of the tumor microenvironment, and their interactions with cancer cells play a major role in mediating the formation and activation of CAFs [16]. In a mouse model of pancreatic insulinoma, it was found that 20–40% of CAFs originate from the bone marrow [17, 18]. Meanwhile, recent data indicated that stimulation of the u-PA/u-PAR system contributes to CAF activation during multiple myeloma (MM) progression [19]. In addition, cancer development depends not only on malignant cancer cells, but also on CAF activation [20]. The crosstalk between cancer cells and CAFs is responsible for cancer cell proliferation, metastasis, invasion, and other critical oncological behaviors [21, 22]. CAFs necessitate targeted treatment, which improves anti-cancer therapy in vitro and animal experiments [23, 24].

Accumulating evidence suggests that miRNAs play a critical role not only in cancer cells but also in the tumor microenvironment [25]. The dysregulation of miRNAs and exosomal miRNAs can influence the crosstalk between cancer cells and the tumor microenvironment [26, 27].

This review focused on the bridging role that exosomal miRNAs play among CAFs, NFs, and cancer cells, exploring how exosomal miRNAs and miRNA dysregulation activate CAFs. We also demonstrated that miRNA dysregulation mediates functional changes in CAFs.

### Exosomal miRNAs constitute a bridge among CAFs, NFs, and cancer cells

Previous studies demonstrated that CAFs and cancer cells regulate each other by secreting a variety of cytokines, chemokines, and extracellular matrix (ECM) [28–31]. However, the mechanism underlying the communication among CAFs, NFs, and cancer cells remains unclear. Recent extracellular vesicle assessments demonstrated that cancer cells communicate with the neighboring cells via soluble factors secreted into the extracellular space [32, 33]. Extracellular vesicles can be classified into three main types according to size and biogenesis: exosomes (30–100 nm), microvesicles (100–1000 nm), and oncosomes (1–10 μm) [34]. Each of these three vesicle types plays crucial roles in cancer biology, with vesicular transport, particularly ‘exosome’ mediated transport standing out [35–37].

Exosomes contain a great variety of bioactive molecules, including signal peptides, microRNAs, lipids, and DNA [38]. A recent study demonstrated that a number of Rab family proteins, including Rab27a and Rab27b, act as key regulators of the exosome secretion pathway [39]. Exosome biogenesis is a very tightly regulated process governed by multiple signaling molecules, and begins with receptor activation that is unique to each cell type [40]. In cancer, tumor cells aberrantly secrete large amounts of exosomes to transport paracrine signals or to contribute to tumor-environment interactions at a distance. Exosomal miRNAs were first identified in human serum, and have also been described in several biological fluids, including saliva, breast milk, and urine [41]. Therefore, EVs containing exosomal miRNAs can regulate tumorigenesis and cancer development by altering the vesicular content and supplying the tumor niche with molecules favoring the progression of oncogenic processes such as proliferation, invasion, cancer stem cell propagation, and even drug resistance [42]. Thus, exosomal miRNAs mediate cell to cell communication and play major roles in the crosstalk between cancer cells and the macro−/microenvironment [43, 44]. More importantly, miRNAs are stably transferred by exosomes [45]. MicroRNAs in plasma exosome can be stably stored under different conditions, indicating that exosomal miRNAs are potential biomarkers or therapeutic tools [46].

#### Exosomal miRNAs and miRNA dysregulation mediate CAF formation and activation

Studies assessing the origin and activation of CAFs have been performed for several years, but the available data remain insufficient. MicroRNAs are dysregulated in several types of cancers [47], modulating cancer proliferation and progression [48, 49]. Recent studies have gradually established connections among exosomal miRNAs, miRNA dysregulation, and CAF activation (Table 1).

**Table 1:** MicroRNAs and exosomal miRNAs mediate CAF formation and activation

Differentially expressed microRNAs	Cancer type	Mechanism	Cancer cell function change	*Ref*
Exosomal miR-155	Pancreatic cancer	Down-regulation of its target TP53INP1	Not mentioned	[50]
Exosomal miR-211	Melanoma cancer	Inhibition of IGF2R, hyper-activating IGF1R/MAPK signaling.	Proliferation, motility, collagen contraction	[55]
Exosomal miR-21/−155/−146a/−148a and let-7 g	Chronic lymphocytic leukemia	Not mentioned	Not mentioned	[52]
Exosomal miR-133b	Prostate cancer	IL-6 stimulation of CAF released miR133b to active NF	Not mentioned	[54]
Exosomal miR-9	Triple-negative breast cancer	Not mentioned	Migration and invasion	[53]
Down-regulated miR-1/−206 and up-regulated miR-31	NSCLC	FOXO3a/VEGFA/CCL2 signaling	Migration, colony formation, TAMs recruitment, tumor growth, and angiogenesis	[56]
Down-regulated miR-31/−214 and up-regulated miR-155	Ovarian cancer	miR-214 target CCL5	Growth, invasion, motility	[57]
Up-regulated miR-210	Prostate cancer	Overexpression of hypoxia-up-regulated miR-210	EMT and angiogenesis	[62]
Up-regulated miR-21	Human normal primary fibroblasts	MiR-21 binds to the 3’UTR of Smad7 mRNA and inhibits its translation to reduce the competition between TGFBR1; Smad 7 binds to Smad 2 and 3.	Not mentioned	[63]
Up-regulated miR-21	Esophageal cancer	Not mentioned	Not mentioned	[64]
Up-regulated miR-27a/b	Esophageal cancer	Not mentioned	Increased secretion of TGF-β leads to cisplatin resistance	[59]
UP-regulated miR-7	Head and neck cancer	Not mentioned	MiR-7 down-regulates RASSF2, which decreases PAR-4 secretion from CAFs, enhancing cell proliferation and migration	[60]
Up-regulated miR-199a/−214	Pancreatic cancer	Not mentioned	Migration and proliferation	[65]
Down-regulated miR-149	Gastric cancer	MiR-149 inhibits the activation of fibroblasts by reducing IL-6 and EP2 expression	EMT and stem-like properties	[66]
Up-regulated miR-409-3p/−409-5p	Prostate cancer	Not mentioned	Over-expressed miR-409 in NFs confers a CAF phenotype and leads to miR-409 release by extracellular vesicles to promote tumorigenesis and EMT	[84]
Down-regulated miR-200 s	Breast cancer	Down-regulated miR-200 s up-regulates fibronectin and lysyl oxidase	Migration, invasion, metastasis	[61]

##### Exosomal miRNAs promote the formation and activation of CAFs

Previous studies demonstrated that pancreatic cancer cells secrete miR-155 to activate NFs. This phenomenon might be related to miR-155-mediated down-regulation of its target TP53INP1 [50]. An innovative study showed that melanoma cells release miR-211-containing melanosomes, which were subsequently taken up by NFs. MicroRNA-211 inhibits the tumor suppressor IGF2R, thereby hyper-activating the IGF1R/MAPK signaling pathway. This promotes CAF formation and melanoma lung metastasis [51]. Exosomes released by chronic lymphocytic leukemia cells induce a CAF-like phenotype in NFs; miR-21, miR-155, miR-146a, miR-148a, and let-7 g are the most abundant miRNAs in these exosomes. However, an in-depth study of these five miRNAs is pending [52].

A study of triple negative breast cancer revealed a CAF-like phenotype inducible by tumor cells through exosome-mediated delivery of miR-9. Interestingly, miR-9 is also released by NFs and transferred to tumor cells. Another study found that miR-9 stimulates tumor cell migration by reducing E-cadherin levels [53]. Doldi et al. [54] reported that IL-6-stimulated CAFs release miR-133b to support paracrine activation of NFs.

##### MicroRNA dysregulation mediates CAF formation and activation

As mentioned above, cancer cells and CAFs release exosomal miRNAs to promote CAF formation [50, 52–55]. Here, we focused on miRNA dysregulation in CAFs in association with their formation and activation.

Our recent study demonstrated that miR-1 and miR-206 down-regulation and miR-31 up-regulation in NFs induce a functional conversion into CAFs and promote the migration, colony formation, and tumor growth of cancer cells, as well as tumor-associated macrophage (TAM) recruitment. Further studies demonstrated that miRNAs reprogram NFs into CAFs by mediating FOXO3a/VEGFA/CCL2 signaling [56]. A similar study in ovarian cancer revealed that miR-214 and miR-31 down-regulation and miR-155 up-regulation induce a functional conversion of NFs into CAFs. This results in a large number of up-regulated genes, highly enriched in chemokines, similar to the gene expression profile of CAFs; the most up-regulated chemokine, CCL5, was found to be a direct target of miR-214 [57, 58].

Another study of esophageal cancer demonstrated that miR-27a/b mediates CAF formation, with increased production of TGF-β leading to cisplatin resistance [59]. It is well-known that over-expression of miR-7 in NFs induces a functional conversion of NFs into CAFs in head and neck cancer (HNC) by downregulating RASSF2, which consequently decreases PAR-4 secretion by CAFs, enhancing the proliferation and migration of the co-cultured cancer cells [60]. Similar findings indicated that NFs, with down-regulated miR-200 s, display traits of activated CAFs in breast cancer and induce cancer cell migration, invasion, and metastasis via fibronectin and lysyl oxidase up-regulation [61]. In prostate cancer, overexpression of hypoxia-induced miR-210 in young fibroblasts increases senescence-associated features and converts these cells into CAF-like cells, thereby promoting EMT in cancer cells [62].

MiR-21 binds to the 3’UTRs of Smad7 mRNA and inhibits its translation. To reduce the competitive binding with TGFBR, Smad7 binds to Smad2, Smad3. Over-expression of miR-21 or Smad7 depletion promotes CAF formation, even without TGF-β1 stimulation [63]. Another study of esophageal cancer also showed that miR-21 might activate NFs and convert them into CAFs [64]. In pancreatic cancer, over-expression of miR-199a and miR-214 is the major regulator of CAF activation, inducing cancer cell migration and proliferation [65]. MiR-149 is down-regulated in gastric cancer CAFs; it inhibits fibroblast activation by down-regulating IL-6. Further studies demonstrated that CAFs enhance EMT and stem-like properties in GC cells in a miR-149-IL-6-dependent manner; PTGER2 (subtype EP2) is also a potential target of miR-149. *Helicobacter pylori* infection potentially promotes the pro-tumor properties of stromal fibroblasts by silencing mmu-mir-149 and stimulating IL-6 production [66]. Interestingly, miR-409 expression in NFs confers a CAF phenotype and results in miR-409 release via extracellular vesicles to promote tumor induction and EMT [31].

The above findings suggested that cancer development depends not only on malignant tumor cells, but also on CAF activation.

#### CAF-secreted exosomal miRNAs affect the functions of cancer cells

**Fig. 1:**
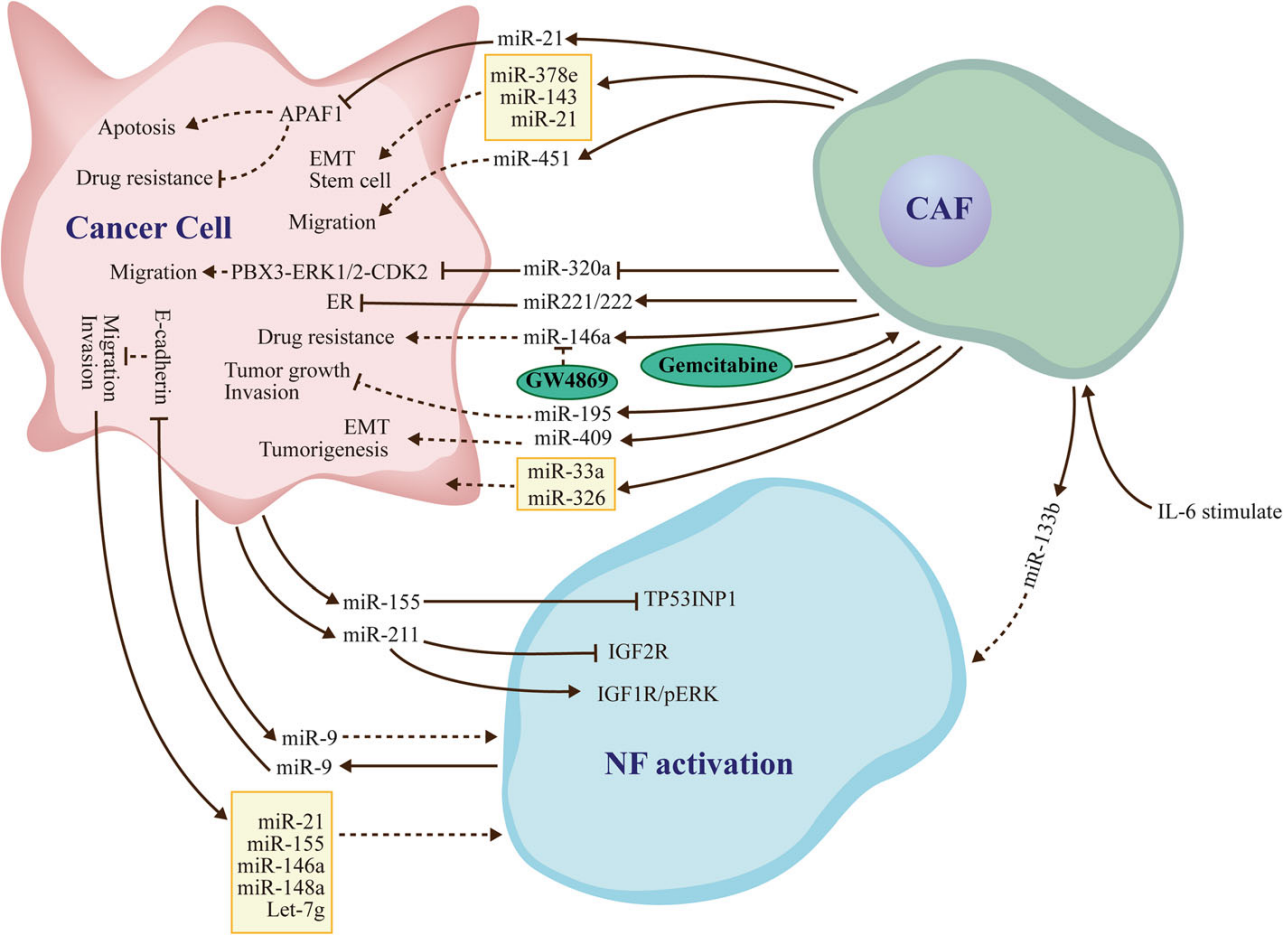
Exosomal miRNAs mediate communication among CAFs, NFs, and cancer cells. Exosomal miRNAs mediate communication in the cell-micro environment and promote the formation of CAFs. Exosomal miRNAs secreted by CAFs and NFs impact migration, invasion, and metastasis in cancer cells, and dictate an aggressive cancer phenotype. Exosomal miRNAs modulate metabolism in cancer cells, and are closely related to drug resistance

Previous findings demonstrated that exosomal miRNAs can be taken up by neighboring or distant cells, subsequently leading to changes in gene expression; this suggests a cell-specialized role in physiological and pathological conditions [67]. Here, we compiled the available literature with respect to exosomal miRNAs associations with the interactions among CAFs, NFs, and cancer cells (Fig. 1).

##### Exosomal miRNAs and miRNA dysregulation lead to drug resistance

Drug resistance is a crucial factor affecting patient prognosis, and attracts increasing attention [68]. Although great efforts have been made to resolve the clinical issue of drug resistance, the underlying mechanism remains largely unclear [69]. Nevertheless, most studies proposed CAFs to be closely associated with chemoresistance acquisition and poor clinical prognosis [70, 71]. Dysregulation of miRNAs in CAFs and exosomal miRNA transfer between cancer cells and the microenvironment is correlated to chemoresistance regulation [72].

A recent study demonstrated that fibroblast-derived exosomes induce cancer stem cells that contribute to chemoresistance [73]. Furthermore, CAFs exposed to gemcitabine increase miR-146a and Snail secretion levels, a feature closely related to gemcitabine resistance. In addition, GW4869, an inhibitor of exosome release, significantly reduces survival in co-cultured epithelial cells [74], suggesting that drug-induced exosome miRNAs may be closely associated with drug resistance after treatment with chemotherapeutic agents. MiR-21 is a widely reported miRNA in several types of tumors. Interestingly, cancer cell-released exosomal miR21 promotes angiogenesis, and is involved in neoplastic processes [75, 76]. Moreover, exosomal miR-21 released by CAFs causes paclitaxel resistance by targeting APAF1 in ovarian cancer and decreasing apoptosis [77].

Dysregulation of miRNAs in CAFs results in drug resistance. The low expression of miR-1 induced CAFs causes high secretion levels of SDF-1α. Meanwhile, SDF-1α facilitates lung cancer cell proliferation and cisplatin resistance via CXCR4-activated NF-κB and Bcl-xL [78]. MicroR-27a/b over-expressed in CAFs can alter esophageal cancer cell sensitivity to cisplatin by increasing TGF-β release [59].

Altered exosomal miRNA profiles during drug administration demonstrates that drug resistance is a complex and dynamic process. In recent years, studies revealed that drug resistance is not only related to genomic or epigenomic changes, but also highly regulated by altered tumor cell metabolism [79]. Targeting the cancer stroma inhibits the metastatic outgrowth, indicating that interference with stromal reorganization might constitute a critical method to prevent recurrent transmitted diseases [80].

##### Exosomal miRNAs influence cancer cell migration, invasion, and metastasis

Exosomal miRNA-regulated cancer biology has been extensively assessed in recent years [81]. However, whether exosomal miRNAs released by CAFs affect cancer cells is relatively understudied. MiR-451, a tumor suppressor, is down-regulated in a variety of tumor types [82]. Conversely, a recent study found that CAFs use exosomal miR-451 as a signaling molecule to promote tumor cell migration and cancer progression [83]. This indicates that miRNA expression levels in tumor cells and CAFs might differ from those in exosomes. MiRNAs might therefore play different roles in cancer cells, CAFs, and exosomes.

Josson et al. [84] found that miR-409 up-regulation in NFs confers a CAF-like phenotype and results in miR-409 release by extracellular vesicles, promoting tumor induction and epithelial-to-mesenchymal transition (EMT). In esophageal cancer, miR-33a and miR-326 are mainly secreted by CAFs, although their functions remain undefined [85]. Interestingly, the antitumor miR-320a is reduced significantly in CAF-derived exosomes of hepatocellular carcinoma patients (HCC). In addition, miR-320a suppresses HCC cell proliferation, migration, and metastasis, by binding to its direct downstream target PBX3 [86]. The miR-320a-PBX3 pathway inhibits the MAPK pathway, which induces epithelial-to-mesenchymal transition and upregulates cyclin-dependent kinase 2 (CDK2) and MMP2 expression in order to promote cell proliferation and metastasis [87].

MiR-195 released by CAFs reduces cancer cell growth and invasion; indeed, miR-195 loaded EVs in rat models of cholangiocarcinoma (CCA) concentrate within the tumor, decrease tumor size, and improve survival in treated animals [88]. These findings suggested that miRNAs transported by exosomes might constitute a therapeutic tool for cancer treatment.

##### Exosomal microRNAs dictate an aggressive cancer phenotype

MiRNA dysregulation often indicates an invasive phenotype in several cancer types through different signaling pathways [89]. Shah et al. [90] demonstrated that CAF-secreted miR221/222 directly suppress ER expression, which is associated with ER-negative breast cancer. In addition, miR-21 and two other miRNAs (378e and 143) are increased in exosomes released from CAFs, and promote the aggressive ability of breast cancer cells, increasing stem cell amounts and inducing EMT [91]. Thus, CAFs strongly promote an aggressive cancer cell phenotype.

##### Exosomal miRNAs modulate cancer cell metabolism

Cancer metabolism alters tumorigenesis, which is vital for sustaining the proliferation and progression of cancer cells in order to support their escape from stringent regulation [92, 93]. Reducing cancer cell metabolism may be a new approach for cancer prevention and treatment [94]. It is known that the altered cancer metabolism is driven by genetic and epigenetic factors [95]. A recent study revealed that microenvironment-secreted exosomes can modulate cancer cell metabolism [96]. In addition, the available literature also confirmed that CAFs secreting exosomal miRNAs modulate cell metabolism in prostate and pancreatic cancers [97].

These findings indicate that exosomal miRNAs play a bridging role in ECM cross-talks, providing deep insights into the communication between CAFs and cancer cells. Thus, targeting circulating miRNAs might be an attractive therapeutic approach for cancer in the future.

### MicroRNA dysregulation mediates functional changes in CAFs

In addition to exosomal miRNA association with interactions among CAFs, NFs, and cancer cells, miRNA dysregulation in CAFs mediates functional changes in CAFs. Most related reports demonstrated that dysregulated miRNAs are associated with tumor invasion, migration, proliferation, metastasis, and drug resistance. In addition, abnormal expression of miRNAs was found to be associated with poor prognosis, and could change the secretory phenotype of CAFs. The retrieved literature was sorted according to functional alterations.

#### MicroRNAs regulate the secretory phenotype of CAFs

As mentioned above, CAFs and cancer cells regulate each other by secreting a variety of cytokines, chemokines, and ECM proteins [28–31]. By altering the secretory phenotype of cancer cells or CAFs, the crosstalk between cancer cells and CAFs can be affected. Recent studies revealed that miRNA dysregulation plays a major role in controlling the secretory function of both cancer cells and CAFs [98]. For instance, miR-335 up-regulated in oral cancer CAFs increases the levels of COX-2 and PGE secreted by CAFs, inducing cancer cell motility by suppressing PTEN expression. MiR-335 is up-regulated by COX-2; conversely, COX-2 inhibition by celecoxib reduces miR-335 expression and restores PTEN production [99]. Similarly, miR-7 overexpression induces a functional conversion of NFs into CAFs through RASSF2 down-regulation, dramatically decreasing PAR-4 secretion from CAFs [60].

**Fig. 2:**
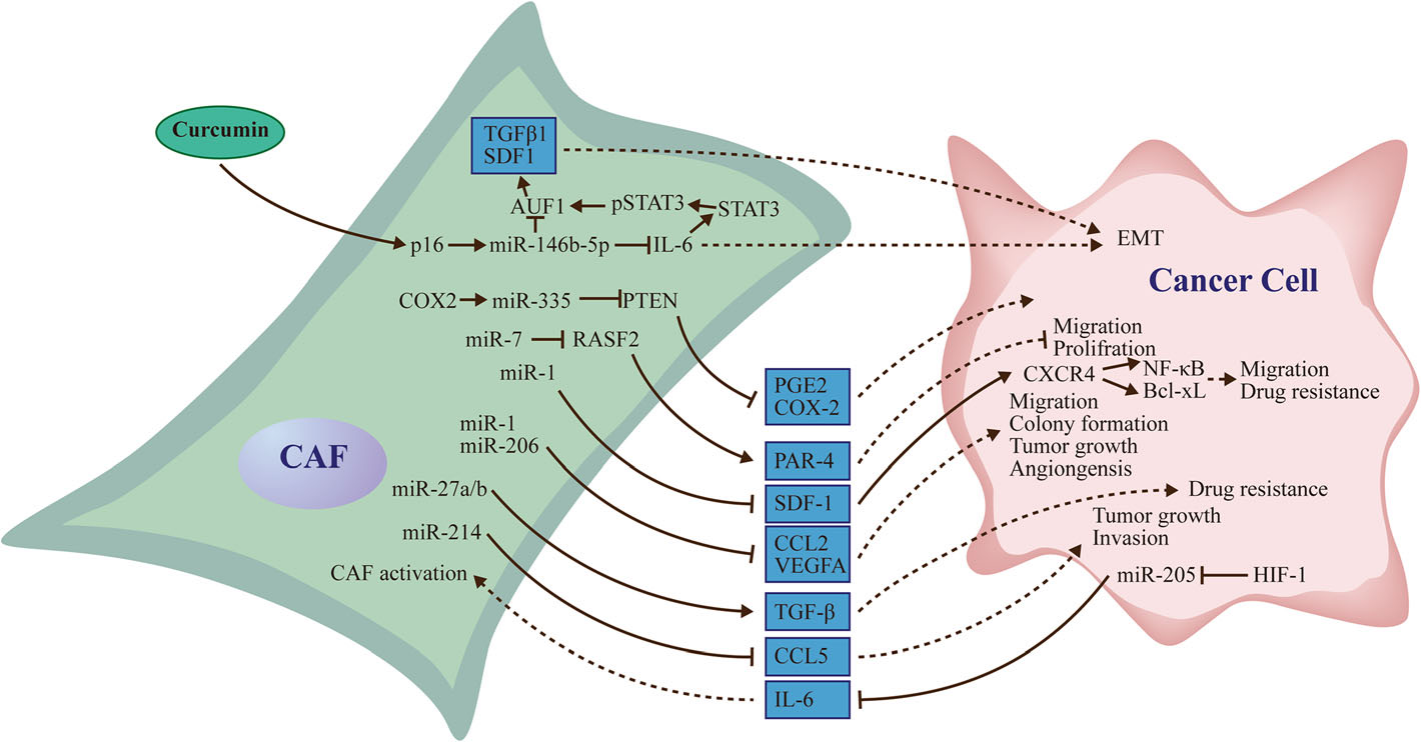
MicroRNAs regulate the secretory phenotype of CAFs and cancer cells. MicroRNAs impact the cross-talk between CAF and cancer cells by modulating the secretory phenotype of CAFs and cancer cells

Another study demonstrated that p16 and miR-146b-5p are down-regulated in breast cancer CAFS; p16 restitution suppresses IL-6 expression and secretion by up-regulating miR-146b-5p in breast cancer [100]. Meanwhile, IL-6 in p16-defective cells is responsible for the paracrine pro-invasive/migratory effects of these cells on breast cancer. Down-regulation of p16 or miR-146b-5p induces a functional conversion of NFs into CAFs [101]. It was shown that curcumin induces p16 and miR-146b-5p, providing a potential treatment option for breast cancer [102]. Down-regulation of miR-1 and miR-206 and up-regulation of miR-31 in NFs induce a functional conversion into CAFs, with increased secretion of CCL2 and VEGFA. Of note, CCL2- and/or VEGFA-neutralizing antibodies administered to BALB/c athymic nude mice bearing pre-established lung tumors drastically reduce tumor growth, angiogenesis, TAM accumulation, and tumor metastasis [56]. Low expression of miR-1 is found in induced CAFs that secrete high levels of SDF-1α. Further studies found that SDF-1α promotes lung cancer cell proliferation and cisplatin resistance via CXCR4-activated NF-κB and Bcl-xL [78]. Similarly, miR-205 is the most down-regulated miRNA in prostate cancer cells as a result of CAF stimulation; miR-205 restitution reduces IL-6 secretion by cancer cells. Another study demonstrated that miR-205 is directly suppressed by HIF-1 [103]. As mentioned above, miR-27a/b mediates CAF formation and induces TGF-β release; in turn, TGF-β alters esophageal cancer sensitivity to cisplatin [59] (Fig. 2).

#### Metastasis

MiR-21, which appears several times in this review, is associated with the formation and activation of CAFs, and exosomal miR-21 released by CAFs can lead to paclitaxel resistance by targeting APAF1 in ovarian cancer [63, 64, 77, 91]. In pancreatic ductal adenocarcinoma, miR-21 is commonly associated with tumor metastasis and closely related to poor prognosis [104]. Clinicopathological findings confirmed that the stromal expression of miR-21 in colorectal cancer patients is closely related to distant metastasis [105].

As mentioned above, down-regulation of miR-200 s predicts a poor outcome in breast cancer patients via elevated expression of Fli-1 and TGF-β, contributing to ECM remodeling and triggering breast cancer cell invasion and metastasis both in vitro and in vivo [61].

#### Tumor migration and invasion

The crosstalk between the matrix and cancer cells determines whether cancer cells remain stable or become invasive and metastatic tumors [106]. MiR-106b is up-regulated in CAFs from gastric cancer, and promotes cell migration and invasion by up-regulating its target PTEN [107]. Conversely, miR-31 is reduced distinctly in endometrial cancer CAFs. Restitution of miR-31 could impair cell migration and invasion by directly targeting the homeobox gene *SATB2*, although cell proliferation is not affected [108]. In ER-positive breast cancer, miR-26b is downregulated in CAFs, leading to enhanced cell migration and invasion [109]. Meanwhile, miR-148a is down-regulated in endometrial cancer CAFs compared with matched NFs; miR-148a restitution directly targets WNT10B, significantly impairing the migration of cancer cells without affecting the growth rate [110]. Down-regulation of miR-148a is considered a marker of metastasis in several other tumors [111, 112]. Similarly, down-regulation of miR-15 and 16 in CAFs promotes prostate tumor proliferation and migration by reducing the post-transcriptional repression of *Fgf-2* and its receptor *Fgfr1* [113]. Moreover, CAFs and NFs regulate migration and invasion in cancer cells through secretory miRNAs [53, 83, 88].

#### MiRNAs and prognosis prediction

Identifying effective prognostic biomarkers is extremely challenging. Interestingly, miR-21 expression in CAFs is associated with decreased overall survival (OS) in pancreatic cancer patients administered 5-FU but not gemcitabine [114]. Naito et al. [115] showed that miR-143 is highly expressed in stromal fibroblasts of scirrhous type gastric cancer, in which it might promote tumor progression by regulating collagen type III through TGF-b/SMAD signaling; multivariate analysis revealed that miR-143 is an independent prognostic factor. A similar study demonstrated that miR-200a is down-regulated in NSCLC CAFs; miR-200a can also down-regulate its target gene hepatocyte growth factor (HGF), and high miR-200a expression is predictive of good prognosis [116]. MicroR-106b expression in CAFs is associated with poor prognosis in gastric cancer [107].

## Conclusion

This review summarized the recent research progress on exosome miRNAs in CAFs, NFs, and cancer cells, highlighting the complex roles of miRNAs in CAFs. First, cancer cells use exosomal miRNAs as signaling molecules to promote the formation of CAFs and modulate the functions of cancer cells. Secondly, miRNA dysregulation is closely related to CAF activation and formation, and affects the tumor-supportive capability of CAFs in vitro and in vivo.

Exosomal miRNAs released by NFs and CAFs can alter migration, invasion, and metastasis in cancer cells, and induce drug resistance. They dictate an aggressive cancer phenotype. MiR-451, a tumor suppressor, is down-regulated in a variety of tumor types; conversely, CAFs use exosomal miR-451 as a signaling molecule to promote tumor cell migration and cancer progression [83]. This also indicated that miRNA expression levels in cancer cells and CAFs might differ from those of exosomes. MicroRNAs may play different roles in cancer cells, CAFs, and exosomes. Exosomal miRNA level changes during drug use also suggest that drug resistance is a complex and dynamic process [74].

Using pre-miRNAs or anti-miRNA inhibitors to restitute the abnormal expression of miRNAs in CAFs can significantly inhibit cancer progression and proliferation in animal models [56]. MiR-195 released by CAFs reduces growth and invasion in cancer cells. Similarly, miR-195 loaded EVs suppress cancer cell proliferation, improving survival as assessed in animal models [88]. CAFs represent a promising target for future cancer therapy, and can resolve the issue of drug resistance. Moreover, injection of miRNAs and miRNA-loaded EVs should be further assessed for cancer treatment.

## Abbreviations

APAF1: apoptotic peptidase activating factor 1
CAFs: Cancer-associated fibroblasts
CCL2: C-C motif chemokine ligand 2
CCL5: C-C motif chemokine ligand 5
CDK2: cyclin-dependent kinase 2
COX2: cytochrome c oxidase subunit II
CXCR4: C-X-C motif chemokine receptor 4
ECM: extracellular matrix
EMT: epithelial to mesenchymal transition
ER: estrogen receptor
EVs: extracellular vesicles
FGF2: fibroblast growth factor 2
FGFR1: fibroblast growth factor receptor 1
FLI1: Fli-1 proto-oncogene
Foxo3a: forkhead box O3A
HCC: hepatocellular carcinoma
HGF: hepatocyte growth factor
HIF1: Hif1p
HNC: head and neck cancer
IGF1R: insulin like growth factor 1 receptor
IGF2R: insulin-like growth factor 2 receptor
IL-6: interleukin 6
MAPK: map kinase
miRNAs: microRNAs
MM: multiple myeloma
MMP2: matrix metallopeptidase 2
NFs: normal fibroblasts
NSCLC: Non-Small Cell Lung Cancer
P16: biomineralization protein SpP16
PAWR (PAR4): pro-apoptotic WT1 regulator
PBX3: PBX homeobox 3
PTEN: phosphatase and tensin homolog
PTGER2: prostaglandin E receptor 2
RASSF2: Ras association domain family member 2
SATB2: SATB homeobox 2
SMAD: SMAD family member
TAMs: Tumor-associated macrophages
TGF-β1: Transforming Growth Factor-β1
TP53INP1: Tumor protein 53-induced nuclear protein-1
UPA: urokinase
UPAR: urokinase receptor
VEGFA: vascular endothelial growth factor A
WNT10B: Wnt family member 10B
